# Elective Initial Blood Testing for the Neurological Outcomes of Pediatric Out‐of‐Hospital Cardiac Arrests

**DOI:** 10.1111/ped.70425

**Published:** 2026-05-06

**Authors:** Kanako Higashi, Soichi Mizuguchi, Noriyuki Kaku, Wakato Matsuoka, Kenichi Tetsuhara, Satoshi Honjo, Tomohiko Akahoshi, Yasunari Sakai, Shouichi Ohga

**Affiliations:** ^1^ Department of Pediatrics, Graduate School of Medical Sciences Kyushu University Fukuoka Japan; ^2^ Emergency and Critical Care Center Kyushu University Hospital Fukuoka Japan; ^3^ Department of Pediatrics Yanagawa Institute for Developmental Disabilities Yanagawa Fukuoka Japan; ^4^ Department of Clinical Research NHO Fukuoka National Hospital Fukuoka Japan

**Keywords:** initial blood tests, neurological outcomes, pediatric OHCA, prognosis, resuscitation

## Abstract

**Introduction:**

Predictive blood testing for outcomes of out‐of‐hospital cardiac arrest (OHCA) remains unclear in childhood.

**Methods:**

We retrospectively analyzed < 15 years old patients with OHCA who entered Kyushu University Hospital between 2006 and 2021. Pediatric Cerebral Performance Category Scale (PCPC) and ΔPCPC score (post‐arrest PCPC 30 days minus pre‐arrest value) classified them into intact survival (ΔPCPC = 0) or poor survival (ΔPCPC = 1–4), and death or brain‐death (PCPC = 6). Initial laboratory data on admission were studied focusing on outcomes and time from detection of CA to emergency room (ED).

**Results:**

Eligible 115 patients included 38 (15 intact‐, 23 poor‐) survivors and 77 deaths. Serum transaminase, lactate dehydrogenase, and potassium levels were higher in death cases than survivors, but did not differentiate poor from intact survivors. Blood gas data showed better levels in intact survivors than others, but did not differentiate poor survivors from deaths. In 41 witnessed cases including 16 (8 intact‐, 8 poor‐) survivors, no one survived with > 40 min of time from detection of CA to ED. In 28 witnessed cases with < 40 min of time from CA to ED, no biochemical variables discriminated the three groups of patients, but three blood gas parameters (pH, HCO_3_^−^, and BE) differentiated intact survivors from the other two groups. Furthermore, pH and BE were correlated with the duration of CA before hospital arrival in the analysis of witnessed OHCA patients.

**Conclusions:**

Initial blood gas data on admission provide valuable information for estimating the duration of CA before hospital arrival and reflect survivors' outcomes in pediatric OHCA patients.

**Abbreviations**
ALT: alanine aminotransferase
AST: aspartate aminotransferase
BE: base excess
CA: cardiac arrest
CK: creatine kinase
CPR: cardiopulmonary resuscitation
ED: emergency department
EMS: emergency medical services
LDH: lactate dehydrogenase
OHCA: out‐of‐hospital cardiac arrest
PCPC: Pediatric Cerebral Performance Category Scale
ROSC: return of spontaneous circulation

## 1 Introduction

The best clinical outcome of pediatric patients who experience out‐of‐hospital cardiac arrest (OHCA) is an intact survival because they have a long‐life expectancy. Previous studies have identified that clinical characteristics such as older age [1, 2], asphyxial cardiac arrest [3], witnessed cardiac arrest [2, 4], bystander cardiopulmonary resuscitation (CPR) [5], and initial shockable rhythm [2, 4] were significantly associated with favorable survival and neurological outcomes in pediatric OHCA. Recently, physiological and imaging data, such as electroencephalography, cerebral oxygen saturation, and computed tomography (CT), have been extensively investigated for their potential to predict the survival and long‐term outcomes of patients with OHCA [6, 7, 8, 9]. However, these studies require considerable time and resources, making them less practical for immediate point‐of‐care in the emergency room (ED). Additionally, CT and magnetic resonance imaging findings after brain injury progress several days after cardiac arrest (CA). Imaging studies thus need the timely start and repeated evaluations [10]. Consequently, these are not effective for rapidly assessing the prognosis of OHCA soon after return of spontaneous circulation (ROSC). Based on no validated early predicting tools (< 48 h after cardiac arrest), current international guidelines including the European Resuscitation Council and the Swedish Resuscitation Council statements recommend at least 72 h of observation and the use of multimodal tools for decision‐making [11, 12].

Initial blood testing obtained upon patient arrival offers a potential avenue for early prognostication. Early prediction of neurological outcomes in survivors with pediatric OHCA helps optimize post‐resuscitation care, guide treatment decisions, and provide appropriate support to the family [13]. Several studies reported associations of serum biochemical data (LDH, AST, CK, and K) [14, 15] or blood gas parameters (pH, pCO_2_, HCO_3_^−^, and BE) [16, 17, 18, 19] with the survival and neurological outcomes in patients with OHCA. However, these studies analyzed data by categorizing patients into (1) survival group and deaths, (2) ROSC and non‐ROSC groups, or (3) neurologically intact survivors and others (poor survivors and deaths). Therefore, there is little information about the predictive utility of initial blood data for neurological outcomes in survivors with pediatric OHCA. Recently, initial blood pH has been reported to be an independent factor for neurological recovery in adult and pediatric patients with OHCA [16, 18, 19]. However, these studies, which included unwitnessed OHCA cases, used “call to hospital arrival time” as a surrogate for CA duration and did not evaluate the association between initial pH values and the actual duration of CA.

To address these limitations, we designed this study with one primary objective—to determine whether initial blood parameters at ED arrival differentiate neurological outcomes among survivors of pediatric OHCA—and two secondary objectives: (1) to examine the association between these parameters and the actual duration of witnessed cardiac arrest, defined as the interval from arrest detection to ED arrival; and (2) to evaluate whether these parameters predict outcomes independently of arrest duration through a subgroup analysis conducted among witnessed OHCA cases.

## 2 Materials and Methods

### 2.1 Study Design and Setting

We conducted a retrospective cohort study of patients with OHCA ≤ 15 years old who consecutively entered the emergency department of Kyushu University Hospital, a tertiary emergency and critical care center in Fukuoka, Japan, between August 1, 2006, and July 31, 2021. This study was approved by the Institutional Review Board of Kyushu University Hospital (IRB approval number: #2022‐083 under the title “An investigation into the associations of initial blood testing with resuscitation time and neurological outcomes in pediatric OHCA patients.” [approved August 3, 2022]). Informed consent was obtained in the form of an opt‐out on a website. All research procedures were conducted in accordance with the local IRB requirements and the amended Helsinki Declaration.

### 2.2 Study Population

This study included all patients with OHCA who reached the ED with ongoing resuscitation by emergency medical services (EMS) and immediately received peripheral blood sampling for both blood gas and biochemical analyses. Both witnessed and unwitnessed cases were included among the subjects, as well as patients who did not achieve ROSC in the ED. The exclusion criteria included ROSC before ED arrival, a lack of initial blood gas and biochemical values, and suspicion of physical abuse due to uncertainty in the medical history, including the CA onset time.

### 2.3 Data Collection

The following patient data were collected from medical and EMS patient care records: age, sex, underlying disease, prodromal symptoms, etiology of cardiac arrest, prehospital resuscitation variables (witness, initial cardiac rhythm, and bystander CPR), and the survival and neurological outcomes 30 days after CA. Initial blood gas (pH, HCO_3_^−^, base excess [BE], and lactate) and biochemical values (alanine aminotransferase [ALT], AST, LDH, CK, potassium, and calcium) at ED arrival were collected from medical records. In witnessed OHCA cases, time from detection of CA to ED was recorded. According to the Pediatric Cerebral Performance Category Scale (PCPC) and ΔPCPC score (PCPC 30 days after cardiac arrest minus PCPC before cardiac arrest), neurological outcomes were categorized into three groups: intact survivor, ΔPCPC = 0; poor survivor, ΔPCPC = 1–4; and brain‐dead or dead (deaths), PCPC = 6.

### 2.4 Outcome Measures

First, as the primary analysis, the clinical profiles and initial blood data obtained at ED arrival were analyzed according to neurological outcomes at 30 days after CA using a three‐category neurological outcome classification (intact survivors, poor survivors, and deaths). For comparison with previous studies, the same clinical profiles and initial blood data obtained at ED arrival were also analyzed according to survival status at 30 days (survivors vs. deaths). Second, in witnessed OHCA cases, we assessed the correlation between the initial blood data and the time from detection of CA to ED arrival and compared the clinical profiles and initial blood data across the same three‐category neurological outcome groups (intact survivors, poor survivors, and deaths). To further explore factors associated with favorable neurological outcomes, a supplementary analysis was performed among witnessed CA patients, comparing intact survivors with patients who had poor neurological outcomes or died. Finally, to examine the predictive value of blood parameters while minimizing variability in CA duration, we performed a subgroup analysis restricted to witnessed OHCA cases with a transport time of less than 40 min.

### 2.5 Statistical Analyses

Differences in the distribution of categorical variables were analyzed using the chi‐square test or Fisher's exact test. Continuous variables were assessed using the Mann–Whitney *U*‐test. Comparisons among the three groups were performed using the Kruskal–Wallis test. Significance was set at *p* < 0.05. In addition, the correlation between the initial blood data and time from detection of CA to ED was assessed using Spearman's rank correlation coefficient (*r* values ranging from −1 to 1) and a simple linear regression analysis. Logistic regression analyses were performed to determine the association between clinical profiles and neurological outcomes. Multivariable logistic regression analysis was performed to identify independent predictors of intact survival. Based on clinical relevance and to minimize model overfitting given the limited sample size, adjustment was performed for age, sex, and no‐flow time. These statistical analyses were performed using the JMP Pro software program (ver. 17.0.0; SAS Institute, Cary, NC, USA).

## 3 Results

### 3.1 Patient Background Characteristics

After excluding 78 patients, 115 were ultimately enrolled as eligible participants (Figure S1). The median age was 27 (range: 0–191) months with male predominance (*n* = 77) (Figure S2). Seventy‐seven patients (67%) died within 30 days after the CA (Figure S1). Fifteen of 38 survivors had no deterioration from baseline 30 days after CA, and the remaining 23 had some recognizable deterioration. There was a significant difference in the etiology, underlying disease, initial rhythm, proportion of witnessed cases, and number of epinephrine doses, but not in other variables, among the three groups of patients (Table 1). Neurologically intact survivors had different etiologies and initial rhythms as well as a higher age of onset, a higher proportion of witnessed cases, and a smaller number of epinephrine doses than poor survivors.

**TABLE 1:** Demographics of the patients.

Clinical and laboratory profiles	Intact survivor *N* = 15	Poor survivor *N* = 23	Deaths *N* = 77	*p*	
				Intact vs. poor vs. deaths	Intact vs. poor
Age (months)					
Median	63	28	22	0.07	**0.021**
Range	6–186	0–181	0–191		
Male, *n* (%)	9 (60.0)	15 (65.2)	53 (68.8)	0.79	0.74
Baseline PCPC score, *n* (%)					
1	8 (53.3)	16 (69.6)	57 (74.0)	0.33	0.33
2	3 (20.0)	5 (21.7)	7 (9.1)		
3	2 (13.3)	0 (0.0)	5 (6.5)		
4	2 (13.3)	2 (8.7)	8 (10.4)		
Etiology, *n* (%)					
Airway obstruction	6 (40.0)	6 (26.1)	9 (11.7)	**< 0.001**	**0.029**
Drowning	1 (6.7)	5 (21.7)	7 (9.1)		
Cardiogenic	8 (53.3)	4 (17.4)	8 (10.4)		
Trauma	0 (0.0)	3 (13.1)	8 (10.4)		
Infection	0 (0.0)	0 (0.0)	3 (3.9)		
Cerebral hemorrhaging	0 (0.0)	0 (0.0)	2 (2.6)		
Unknown	0 (0.0)	5 (21.7)	40 (51.9)		
Underlying disease, *n* (%)					
Chromosomal aberration	2 (13.3)	2 (8.7)	5 (6.5)	**0.011**	0.07
Neuromuscular disease	1 (6.7)	1 (4.4)	7 (9.1)		
Cardiac disease	7 (46.7)	3 (13.1)	8 (10.4)		
Others	3 (20.0)	4 (17.4)	7 (9.1)		
None	2 (13.3)	13 (56.5)	50 (64.9)		
Witnessed, *n* (%)	8 (53.3)	8 (34.8)	25 (32.5)	**0.028**	**0.039**
Bystander CPR, *n* (%)	13 (86.7)	14 (60.9)	51 (66.2)	0.22	0.09
Initial rhythm, *n* (%)					
Shockable	7 (46.7)	2 (8.7)	2 (2.6)	**< 0.001**	**0.016**
Asystole	2 (13.3)	10 (43.5)	53 (68.8)		
PEA	6 (40.0)	11 (47.8)	22 (28.6)		
Prodromal symptoms, *n* (%)	6 (40.0)	5 (21.7)	28 (36.8)	0.36	0.23
Number of epinephrine doses, median, range	0, 0–6	2, 0–10	5, 0–10	**< 0.001**	**0.004**

*Note:* Values in parentheses are the percentage. Bold values indicate statistically significant differences (*p* < 0.05).

Abbreviations: CPR, cardiopulmonary resuscitation; PCPC, pediatric cerebral performance category; PEA, pulseless electrical activity.

### 3.2 Initial Blood Tests and Survival Outcomes

The initial biochemical and blood gas data were compared between survivors and deaths. All parameters, except for serum calcium levels, showed significant differences (Figure S3a,b).

### 3.3 Initial Blood Tests and Neurological Outcomes

**FIGURE 1:**
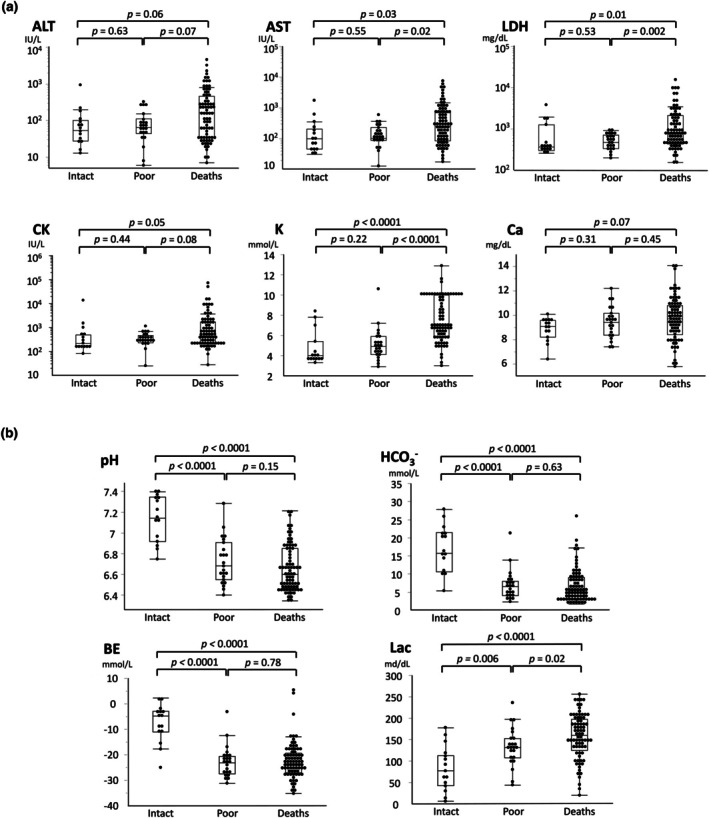
Initial blood data in the three neurological outcome groups. Initial biochemical (a) and blood gas values (b) were compared between the two groups. Data are shown as the median (line), upper and lower quartiles (box), and range (whiskers). The Mann–Whitney *U*‐test *p*‐values are shown above the bars.

The median values of serum AST, LDH, and potassium were significantly higher in death cases than in neurologically intact and poor survivors (Figure 1a). The median CK levels in death cases tend to be higher than those in neurologically intact survivors (*p* = 0.05) but not in poor survivors. There were no significant differences in ALT or calcium levels among the three groups of patients. No biochemical tests discriminated intact from poor survivors. In contrast, all blood gas parameters (pH, HCO_3_^−^, BE, and lactate levels) showed significant differences between intact and poor survivors (Figure 1b). There was a significant decrease in the proportion of intact survivors with decreasing pH levels (Figure S4). Lactate levels alone discriminated death cases from poor survivors.

### 3.4 Background Characteristics of Witnessed Cases

**FIGURE 2:**
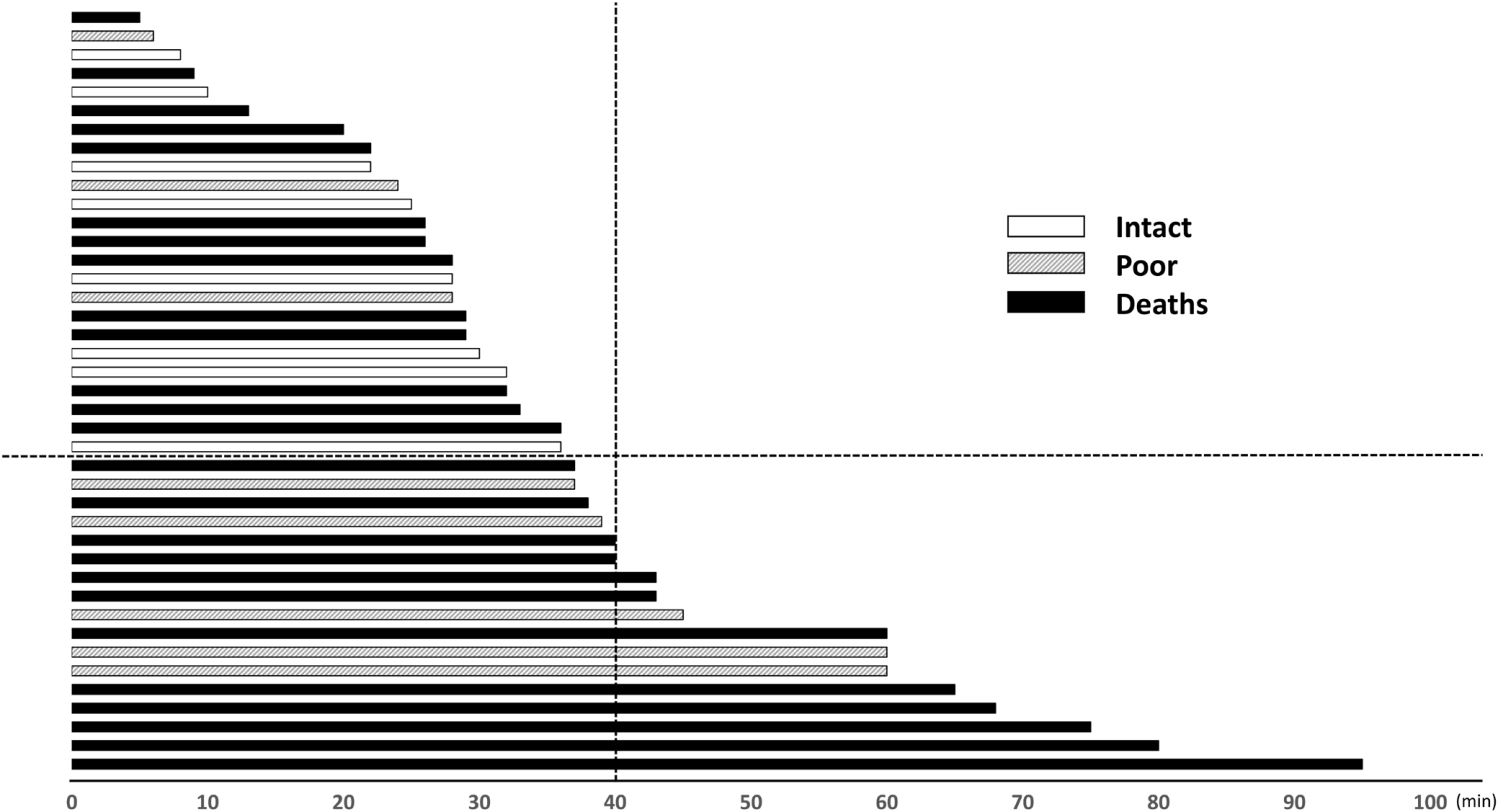
Time from detection of CA to ED of the witnessed OHCA patients. The 41 witnessed OHCA patients were listed in the order of shortest time from detection of CA to ED. The neurological outcomes 30 days after cardiac arrest are shown for intact survivors (plain bars), poor survivors (cross‐hatching bars), and deaths (black bars). All eight intact survivors were listed above the horizontal dotted line and had time from detection of CA to ED of < 40 min (vertical dotted line). OHCA: Out‐of‐hospital cardiac arrest.

Of 115 OHCA patients, 41 were witnessed by bystanders (Figure S5). The median age of the witnessed cases was 44 (range: 0–190) months with male predominance (*n* = 31). Sixteen survivors were neurologically intact and poor in half and half. The remaining 25 died within 30 days after CA (Figure S5). All 8 intact survivors had a time from detection of CA to ED of < 40 min (Figure 2). Univariate analysis showed only an association between airway obstruction and intact survivors. Furthermore, a multivariable analysis, adjusting for sex, age, and no flow time, confirmed these results (Table S1). In the 41 witnessed patients, there was a significant difference in the number of epinephrine doses, but not in other variables, among the three groups of patients (Table S2).

### 3.5 Correlation Between Initial Blood Tests and Detection of CA to ED Time

**FIGURE 3:**
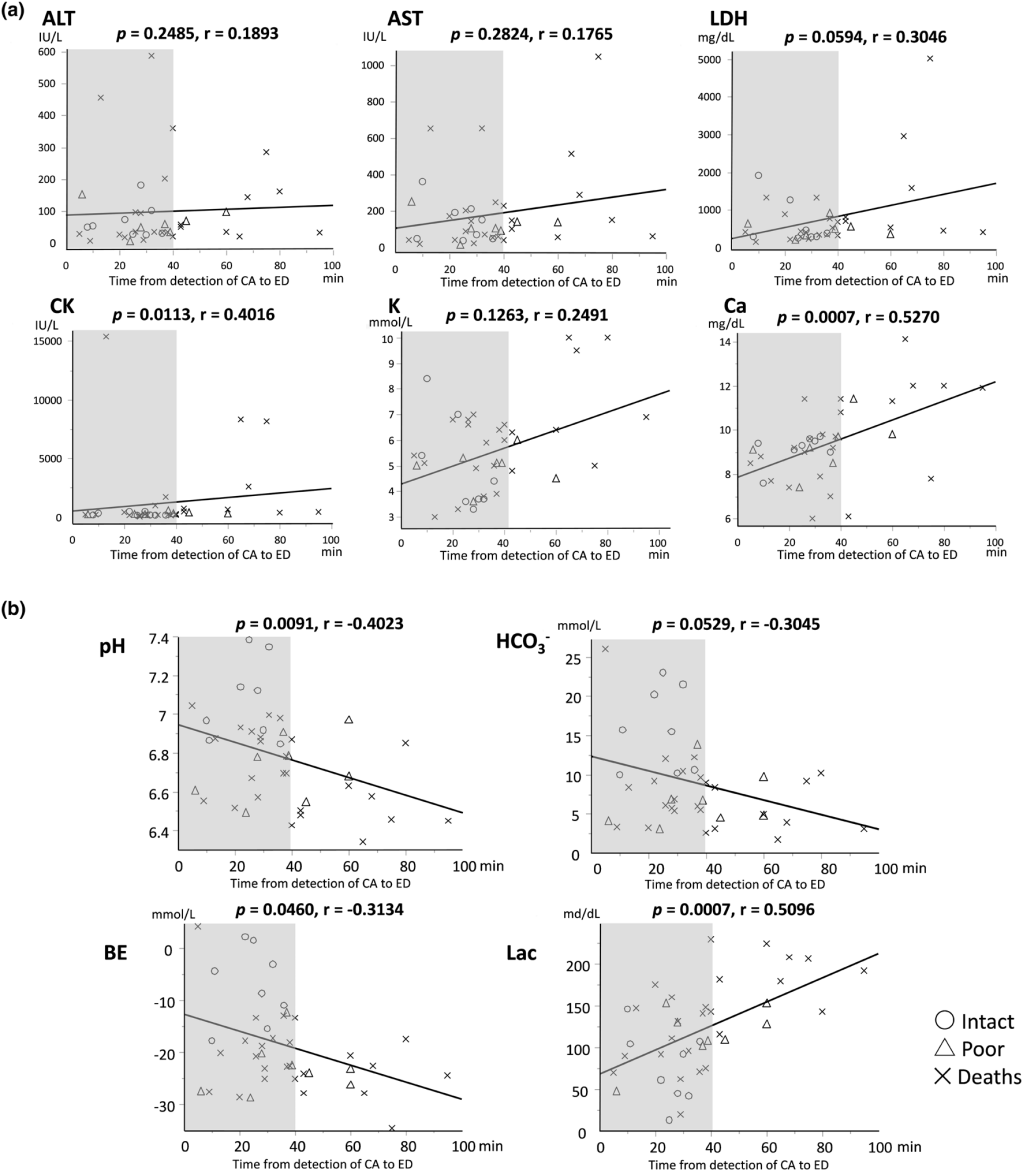
A correlative analysis between initial blood data and time from detection of CA to ED in witnessed OHCA patients. The correlation between initial blood data and time from detection of CA to ED in 41 patients with witnessed OHCA (a: Biochemical values; b: Blood gas values). Each dot represents an individual patient (intact survivors, open circles; poor survivors, closed circles; dead, cross‐marks). The gray area includes patients with time from detection of CA to ED of < 40 min. The regression line, Spearman's correlation coefficient, and *p*‐values are shown in each panel. OHCA: Out‐of‐hospital cardiac arrest.

Three of four blood gas parameters of pH, BE, and lactate levels were correlated with time from detection of CA to ED. Only two biochemical parameters of CK and calcium were associated with time from detection of CA to ED (Figure 3a,b).

### 3.6 Initial Blood Tests and Neurological Outcomes in Witnessed Cases

In the 41 witnessed patients, no biochemical test results differed among the three groups (Table S6a). In contrast, all four blood gas parameters of high pH, HCO_3_^−^, or BE levels and low lactate levels discriminated intact survivors from poor survivors or death cases (Table S6b).

### 3.7 Subgroup Analysis: Witnessed OHCA With Detection of CA to ED Time of < 40 Min

**FIGURE 4:**
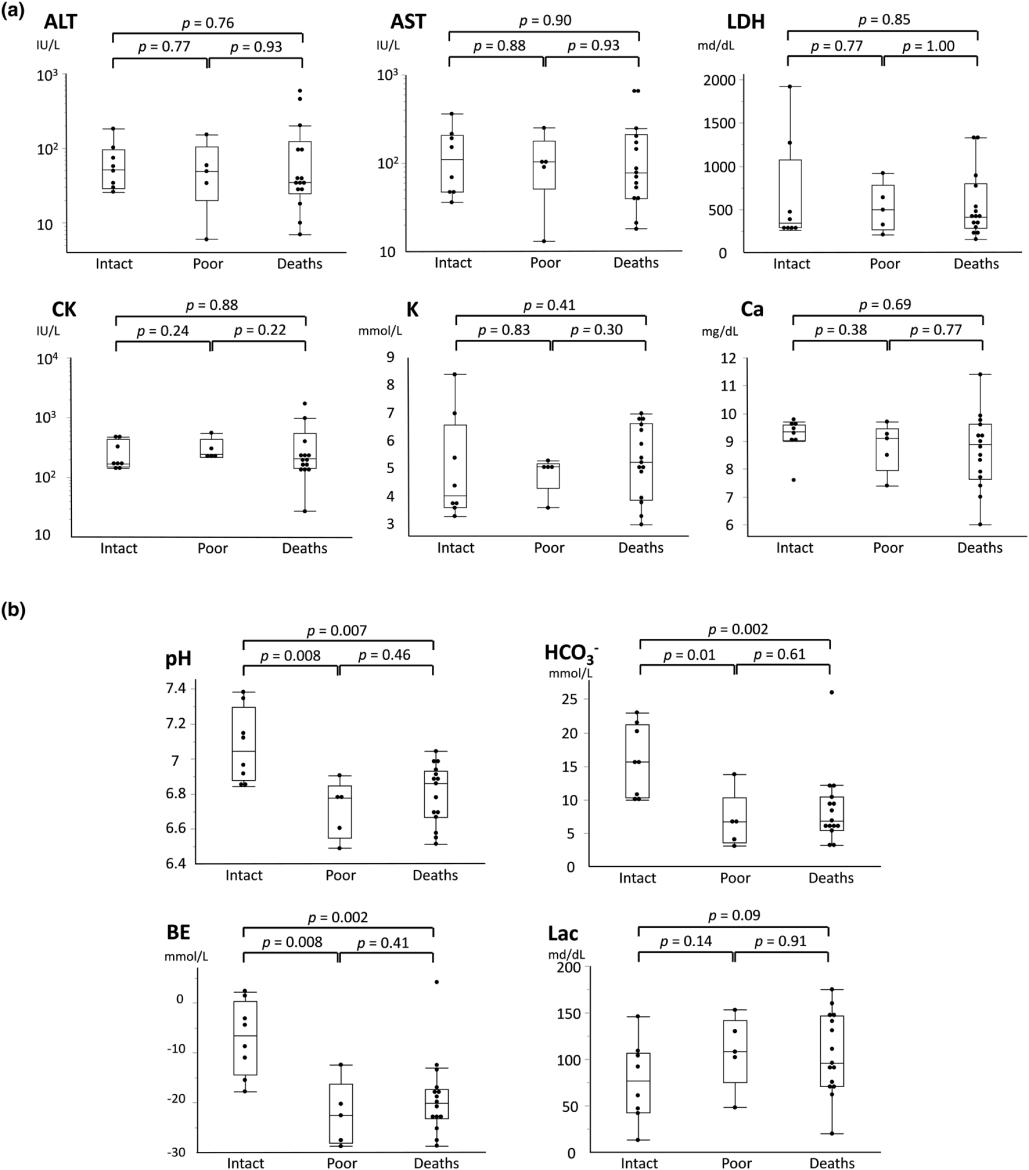
Initial blood data of witnessed OHCA patients with time from detection of CA to ED of < 40 min. Initial biochemical (a) and blood gas values (b) were compared between the two outcome groups. Data are shown as the median (line), upper and lower quartiles (box), and range (whiskers). The Mann–Whitney *U*‐test *p*‐values are shown above the bars.

To further investigate the impact of initial blood tests on the intact survival, we performed subanalysis in witnessed cases with detection of CA to ED time of < 40 min. The biochemical data did not differ markedly between each of the two groups (Figure 4a). The blood gas parameters of pH, HCO_3_^−^, and BE, but not lactate, clearly differentiated intact survivors from the other two groups of patients (Figure 4b).

## 4 Discussion

This is the first study to analyze the association of initial blood test results with the duration of CA before hospital arrival and neurological outcomes in pediatric OHCA survivors. The duration of CA was associated with pH, BE, lactate, CK, and calcium levels. In contrast, the ΔPCPC scoring‐based analysis on the groups of patients found that initial pH, HCO_3_^−^ and BE, but not biochemical data, is useful in distinguishing intact from poor survivors.

Regarding neurological outcomes, this study differs from previous research by categorizing pediatric patients with OHCA into three distinct groups based on their outcomes 30 days post‐arrest: intact survivors, poor survivors, and deaths. Analysis of the intact and poor survivor groups indicated that blood gas parameters were strongly associated with neurological outcomes, while biochemical parameters did not show the same association. Conversely, consistent with previous studies [14, 19], when patients were classified as survivors or non‐survivors, significant differences emerged in all biochemical and blood gas parameters, except for calcium levels.

Resuscitation time is one of the most critical factors affecting the survival and long‐term outcomes of OHCA [20, 21, 22, 23]. Analyses involving CA duration were restricted to witnessed cases. Notably, in patients with witnessed OHCA, all intact survivors were transported in less than 40 min. In subgroup analysis, initial blood testing of patients transported in less than 40 min also showed significant differences in the levels of pH, HCO_3_^−^, and BE between intact and poor survivors. These findings suggest that, in witnessed OHCA cases with prompt transport to the hospital, the identified blood gas parameters indicate differences in patients' condition during CPR that related to neurological outcomes, rather than simply reflecting differences in the time from detection of CA to ED. Okada et al. [16] conducted an extensive analysis using a Japanese registry of pediatric patients with OHCA. They found that pediatric patients with an initial pH value of ≥ 6.82 had better survival rates and neurological outcomes 1 month after CA than those with an initial pH value of < 6.82. However, their study did not include data on the time from detection of CA to ED. The low initial pH has been discussed as reflecting inadequate “cerebral perfusion volume” or “ventilation” during chest compressions [16]. In addition to the clinical backgrounds of pediatric patients with OHCA, such as age, etiology, presence of witnesses, bystander CPR, and initial rhythm [1, 2, 3, 4, 5], pH and BE levels may provide clinically useful information for post‐ROSC management, estimation of CA duration, and assessment of CPR quality.

Among the four parameters of initial blood gas testing, only lactate levels did not show marked differences between intact and poor survivors in witnessed OHCA cases transported within 40 min (Figure 4b). Previous studies have shown that lactate measured later after ROSC or its clearance, rather than initial levels, is associated with outcomes [24, 25], and evidence regarding lactate during CPR remains inconsistent [26, 27, 28]. When the time from detection of CA to ED was brief, as described in this study, elevated lactate levels were low in number among both intact and poor survivors. These findings suggest that pH and BE, unlike lactate levels, serve as an acute indicator of impaired tissue perfusion in association with neurological outcomes in surviving patients.

Pediatric OHCA is associated with a high mortality rate, and even in surviving cases, the functional status is often severely compromised [29]. A recent study has identified withdrawal of life‐sustaining treatment (WLST) based on expected poor neurological outcomes as a prominent cause of death in OHCA patients in the pediatric intensive care unit (PICU) [30]. Despite these challenges, reliable tools for early neurological prognostication in pediatric patients remain lacking. Current international guidelines recommend at least 72 h of observation and the use of multimodal tools for WLST decisions, noting that there are no reliable clinical signs or tests that predict poor neurological outcomes prior to this point [11, 12]. The significance of this study lies in its potential to help clinicians estimate the risk of poor neurological outcomes in pediatric OHCA survivors early and objectively, even before PICU admission. Such early information may assist clinicians in making more informed decisions and facilitate timely, appropriate communication with families regarding expected outcomes.

### 4.1 Limitations

Several limitations associated with the present study warrant mention. First, this was a single‐center retrospective analysis with a limited number of cases. Second, significant differences were identified in etiology and underlying disease status among the three groups, and these variances may have biased the study results. For example, asphyxial arrest is associated with characteristic blood gas patterns during resuscitation [31], whereas traumatic OHCA often results from sudden catastrophic events in which collapse‐to‐EMS time is short [29] and metabolic derangements may be less pronounced despite the generally poor prognosis [32]. Third, as this study assessed neurological outcomes using ΔPCPC, intact survivors included patients with a poor baseline PCPC. To establish a clearer association between initial blood testing, duration of CA, and neurological outcomes in surviving patients, it is necessary to investigate non‐traumatic OHCA patients without underlying diseases on a larger scale and in a multi‐institutional setting. External validation will also be essential to determine whether pH and other initial blood parameters retain their prognostic performance across different institutions and populations.

## 5 Conclusion

The results of the initial blood testing upon ED arrival, especially the levels of pH and BE, are informative for estimating the time from detection of CA to ED and predicting the neurological outcomes of pediatric OHCA. Early prediction of neurological outcomes based on the results of initial blood testing may contribute to supporting appropriate decision‐making and realizing individualized post‐resuscitation care.

## Author Contributions

K.H., S.M., and S.O. designed the study; K.H., S.M., N.K., and W.M. collected and analyzed data; S.H. provided advice in statistical analysis; K.H., S.M., N.K., and W.M. wrote the manuscript; K.T., S.H., T.A., Y.S., and S.O. edited and reviewed the manuscript; S.O. supervised the whole study process. All authors read and approved the final manuscript.

## Funding

This work was supported in part by JSPS KAKENHI Grant numbers JP21K11215 (Wakato Matsuoka) and JP23K15618 (Soichi Mizuguchi).

## Disclosure

The authors have nothing to report.

## Ethics Statement

This study was conducted in compliance with the institutional guidelines for clinical research. The specific protocol for the retrospective analysis was approved by the Institutional Review Board of Kyushu University Hospital (#2022‐083). Informed consent was obtained in the form of an opt‐out option on a website.

## Conflicts of Interest

The authors declare no conflicts of interest.

## Supporting information


**Figure S1:** Flowchart of OHCA patients enrolled in this study.
**Figure S2:** Age distribution of the enrolled patients.
**Figure S3:** Initial blood data in survival outcome groups.
**Figure S4:** Proportion of intact survivors by each pH group.
**Figure S5:** Flowchart of the witnessed OHCA patients enrolled in the present study.
**Figure S6:** Initial biochemical data of the witnessed OHCA patients.
**Table S1:** Univariate and multivariable analysis of witnessed arrest patients to differentiate intact survival from two other group patients.
**Table S2:** Demographics of the witnessed cardiac arrest patients.
