# Supplementary material for: Elective Initial Blood Testing for the Neurological Outcomes of Pediatric Out‐of‐Hospital Cardiac Arrests
Source: Pediatr Int. 2026 May 6;68(1):e70425. doi: 10.1111/ped.70425 (PMC13150397; doi:10.1111/ped.70425)
Supplement: Supplementary file 1 — Figure S1: Flowchart of OHCA patients enrolled in this study. Figure S2: Age distribution of the enrolled patients. Figure S3: Initial blood data in survival outcome groups. Figure S4: Proportion of intact survivors by each pH group. Figure S5: Flowchart of the witnessed OHCA patients enrolled in the present study. Figure S6: Initial biochemical data of the witnessed OHCA patients. Table S1: Univariate and multivariable analysis of witnessed arrest patients to differentiate intact survival from two other group patients. Table S2: Demographics of the witnessed cardiac arrest patients. [file PED-68-e70425-s001.docx]

***Pediatrics International***

**Supplementary materials**

**Elective initial blood testing for the neurological outcomes of pediatric out-of-hospital cardiac arrests**

Kanako Higashi, MD, Soichi Mizuguchi, MD, PhD, Noriyuki Kaku, MD, PhD, Wakato Matsuoka, MD, Kenichi Tetsuhara, MD, PhD, Satoshi Honjo MD, PhD, Tomohiko Akahoshi, MD, PhD, Yasunari Sakai MD, PhD, Shouichi Ohga, MD, PhD

**Corresponding author:** Soichi Mizuguchi, MD, PhD, mizuguchi.soichi.083@m.kyushu-u.ac.jp

Department of Pediatrics, Graduate School of Medical Sciences, Kyushu University

This file contains the following materials:

- Supplementary tables (Table 1, 2)
- Supplementary figures and legends (Fig. 1 - 6)

**Supplementary Table 1. Univariate and multivariable analysis of witnessed arrest patients to differentiate intact survival from two other group patients**

|  |  | Intact survival | Poor survival or dead | Crude odds ratio | 95% CI | | | *P-*value |  | Adjusted odds ratio* | 95% CI | | | *P-*value |
| --- | --- | --- | --- | --- | --- | --- | --- | --- | --- | --- | --- | --- | --- | --- |
|  |  |  |  |  | LL |  | UL |  |  |  | LL |  | UL |  |
| Shockable rhythm | YES | 3 | 4 | 4.4 | 0.7 |  | 25.6 | 0.10 |  | 2.5 | 0.3 |  | 18.5 | 0.37 |
|  | NO | 5 | 29 |  |  |  |  |  |  |  |  |  |  |  |
|  |  |  |  |  |  |  |  |  |  |  |  |  |  |  |
| Airway obstruction | YES | 5 | 3 | 16.7 | 2.6 |  | 107.0 | **0.003** |  | 14.2 | 1.9 |  | 106.0 | **0.01** |
|  | NO | 3 | 30 |  |  |  |  |  |  |  |  |  |  |  |
|  |  |  |  |  |  |  |  |  |  |  |  |  |  |  |
| No flow time 0 min** | YES | 4 | 10 | 2.3 | 0.5 |  | 11.6 | 0.30 |  | 2.7 | 0.5 |  | 14.8 | 0.27 |
|  | NO | 4 | 23 |  |  |  |  |  |  |  |  |  |  |  |
|  |  |  |  |  |  |  |  |  |  |  |  |  |  |  |
| Female** | YES | 4 | 6 | 4.5 | 0.9 |  | 24.7 | 0.07 |  | 5.3 | 0.9 |  | 31.0 | 0.06 |
|  | NO | 4 | 27 |  |  |  |  |  |  |  |  |  |  |  |
|  |  |  |  |  |  |  |  |  |  |  |  |  |  |  |
| 12 months or older** | YES | 7 | 23 | 3.0 | 0.3 |  | 28.1 | 0.33 |  | 3.3 | 0.3 |  | 33.0 | 0.30 |
|  | NO | 1 | 10 |  |  |  |  |  |  |  |  |  |  |  |

ORs (95% confidence intervals) and *P-*values were obtained via a logistic regression analysis.

OR, Odds ratio. CI, Confidence interval. LL, Lower limit. UL, Upper limit

*Adjusted values were obtained via a logistic regression analysis after adjusting for the gender, age, and no flow time.

**Adjusted values were obtained via a logistic regression analysis after adjusting for two factors from among the gender, age, and no flow time.

**Supplementary table 2.** **Demographics of the witnessed cardiac arrest patients**

| Clinical and laboratory profiles | Intact survivor  N = 8 | Poor survivor  N = 8 | Deaths  N = 25 | *P-*value | |
| --- | --- | --- | --- | --- | --- |
|  |  |  |  | Intact vs. Poor vs. Deaths | Intact vs. Poor |
| Age (months) |  |  |  |  |  |
| median | 59 | 43 | 37 | 0.77 | 0.79 |
| range | 11–165 | 8–181 | 0–190 |  |  |
| Male, n (%) | 4 (50.0) | 5 (62.5) | 22 (88.0) | 0.06 | 0.61 |
| Baseline PCPC score, n (%) |  |  |  | 0.15 | 0.17 |
| 1 | 2 (25.0) | 6 (75.0) | 18 (72.0) |  |  |
| 2 | 3 (37.5) | 1 (12.5) | 5 (20.0) |  |  |
| 3 | 2 (25.0) | 0 (0.0) | 2 (8.0) |  |  |
| 4 | 1 (12.5) | 1 (12.5) | 0 (0.0) |  |  |
| Etiology, n (%) |  |  |  | 0.07 | 0.12 |
| Airway obstruction | 5 (62.5) | 1 (12.5) | 2 (8.0) |  |  |
| Drowning | 0 (0.0) | 1 (12.5) | 5 (20.0) |  |  |
| Cardiogenic | 3 (37.5) | 4 (50.0) | 5 (20.0) |  |  |
| Trauma | 0 (0.0) | 2 (25.0) | 6 (24.0) |  |  |
| Infection | 0 (0.0) | 0 (0.0) | 2 (8.0) |  |  |
| Cerebral hemorrhaging | 0 (0.0) | 0 (0.0) | 2 (8.0) |  |  |
| Unknown | 0 (0.0) | 0 (0.0) | 3 (12.0) |  |  |
| Underlying disease, n (%) |  |  |  | 0.05 | **0.04** |
| Chromosomal aberration | 2 (25.0) | 0 (0.0) | 1 (4.0) |  |  |
| Neuromuscular disease | 0 (0.0) | 1 (12.5) | 1 (4.0) |  |  |
| Cardiac disease | 3 (37.5) | 3 (37.5) | 5 (20.0) |  |  |
| Others | 3 (37.5) | 0 (0.0) | 3 (12.0) |  |  |
| None | 0 (0.0) | 4 (50.0) | 15 (60.0) |  |  |
| Bystander CPR, n (%) | 7 (87.5) | 6 (75.0) | 15 (60.0) | 0.31 | 0.52 |
| Initial rhythm, n (%) |  |  |  | 0.25 | 0.84 |
| Shockable | 3 (37.5) | 2 (25.0) | 2 (8.0) |  |  |
| Asystole | 2 (25.0) | 2 (25.0) | 13 (52.0) |  |  |
| PEA | 3 (37.5) | 4 (50.0) | 10 (40.0) |  |  |
| Prodromal symptoms, n (%) | 3 (37.5) | 2 (25.0) | 9 (36.0) | 0.83 | 0.59 |
| Number of epinephrine doses, median, range | 0, 0-2 | 6, 2-10 | 5, 0-10 | **<0.001** | **0.001** |
| No flow time (min),  median, range | 1, 0-13 | 4, 0-20 | 8, 0-69 | 0.13 | 0.44 |
| Time from detection of CA to ED (min), median, range | 27, 8-36 | 38, 6-60 | 36, 5-95 | 0.12 | 0.11 |

Values in parentheses are the percentage.

PCPC, Pediatric Cerebral Performance Category. CPR, Cardiopulmonary resuscitation. PEA, Pulseless electrical activity.

**Supplementary Fig. 1 Flowchart of OHCA patients enrolled in the present study**

After excluding 78 patients, 115 were ultimately enrolled as eligible participants. Fifteen of the 38 survivors had no neurological deficits 30 days after cardiac arrest (CA), and the remaining 23 had some recognizable deficits. Seventy-seven patients died within 30 days after the CA.

PCPC, Pediatric Cerebral Performance Category; OHCA, out-of-hospital cardiac arrest.

**Supplementary Fig. 2** **Age distribution of the enrolled patients**

The graph shows the age distribution of the enrolled patients. Their median age was 27 (range: 0-191) months old with male predominance (n=77).

**Supplementary Fig. 3 Initial blood data in survival outcome groups**


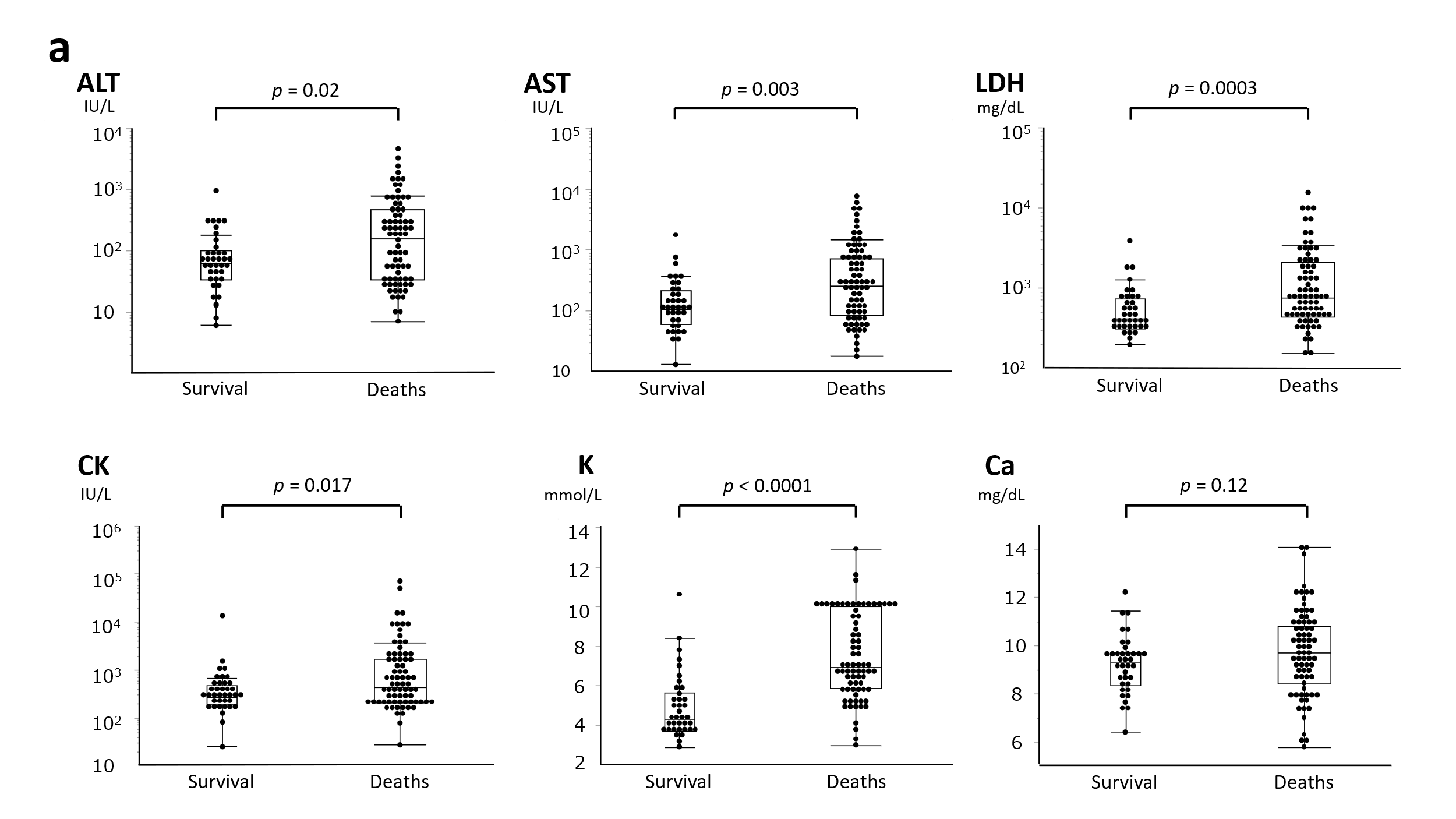


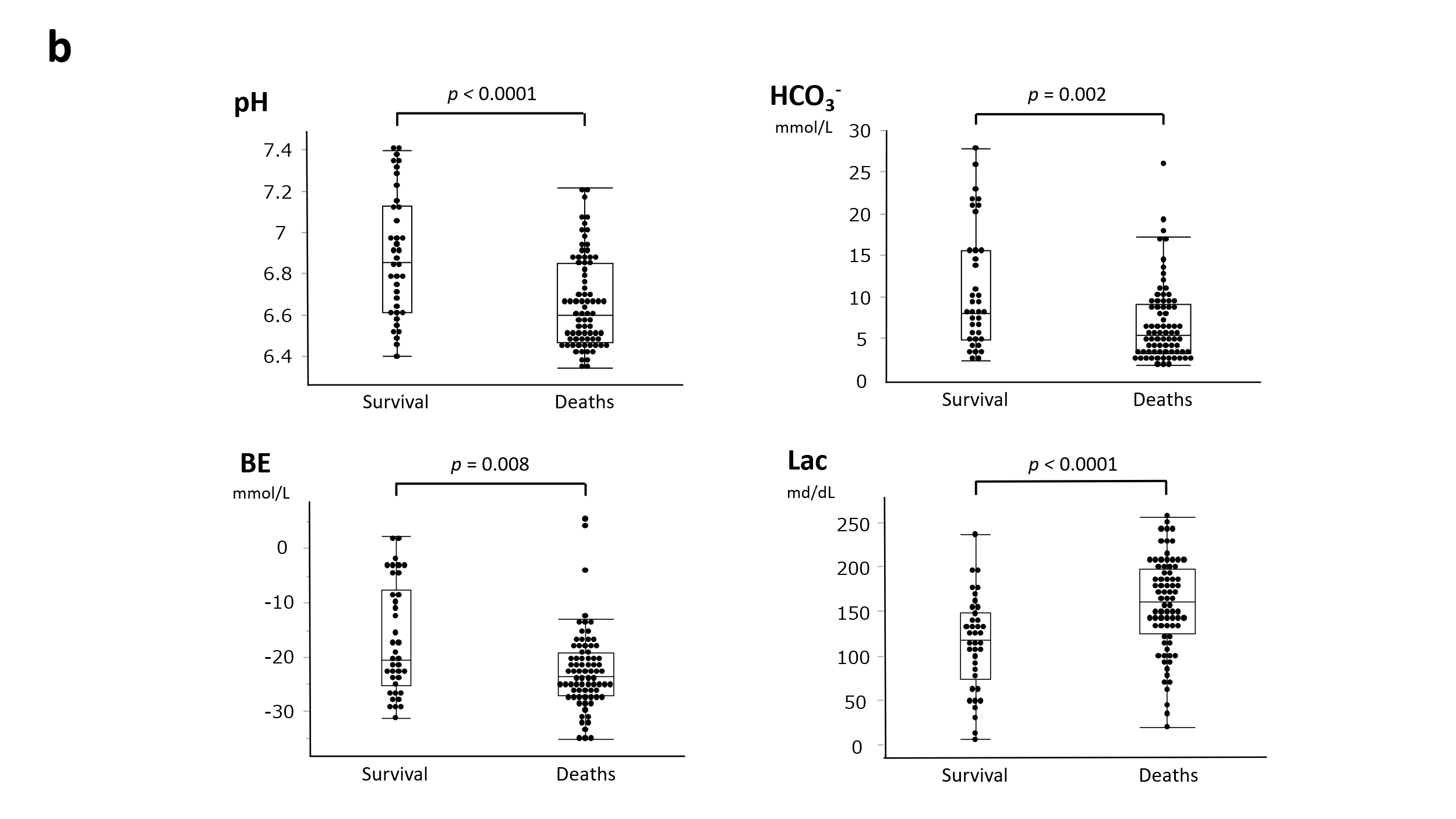


Initial biochemical (a) and blood gas values (b) were compared between survivors and non-survivors at 30 days after cardiac arrest. Data are shown as the median (line), upper and lower quartiles (box), and range (whiskers). The Mann-Whitney U-test p-values are shown above the bars.

**Supplementary Fig. 4 Proportion of intact survivors by each pH group**


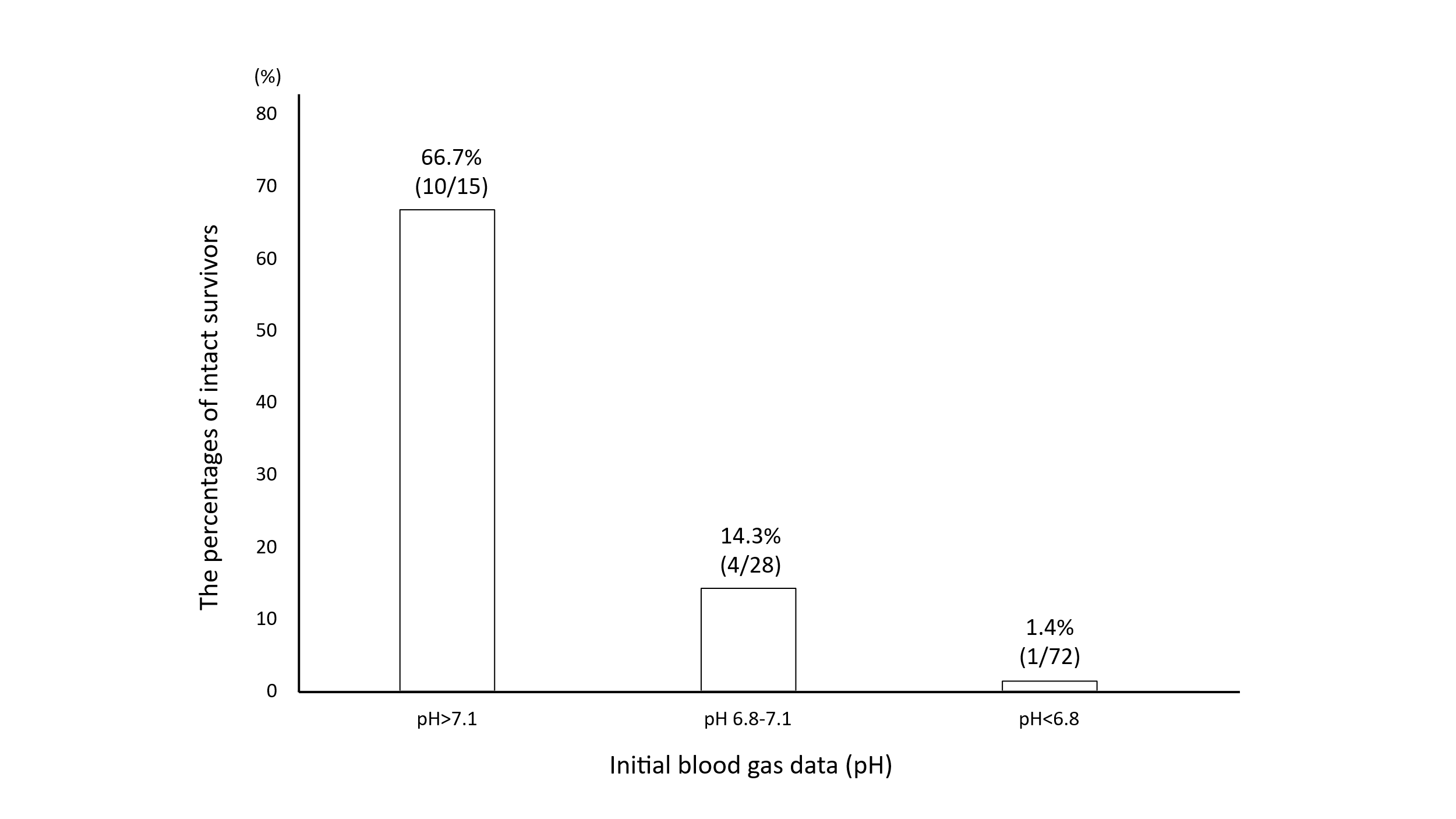


The figure shows the percentage of intact survivors in each pH group: 66.7% (10/15) for pH > 7.1, 14.3% (4/28) for pH 6.8–7.1, and 1.4% (1/72) for pH < 6.8.

**Supplementary Fig. 5** **Flowchart of the witnessed OHCA patients enrolled in the present study**

Of the 115 eligible OHCA patients, 41 were witnessed by bystanders. Of the 16 survivors, half were neurologically intact, and the other half were poor survivors. The remaining 25 patients died within 30 days after the CA.

PCPC, Pediatric Cerebral Performance Category; OHCA, out-of-hospital cardiac arrest.

**Supplementary Fig. 6 Initial biochemical data of the witnessed OHCA patients**


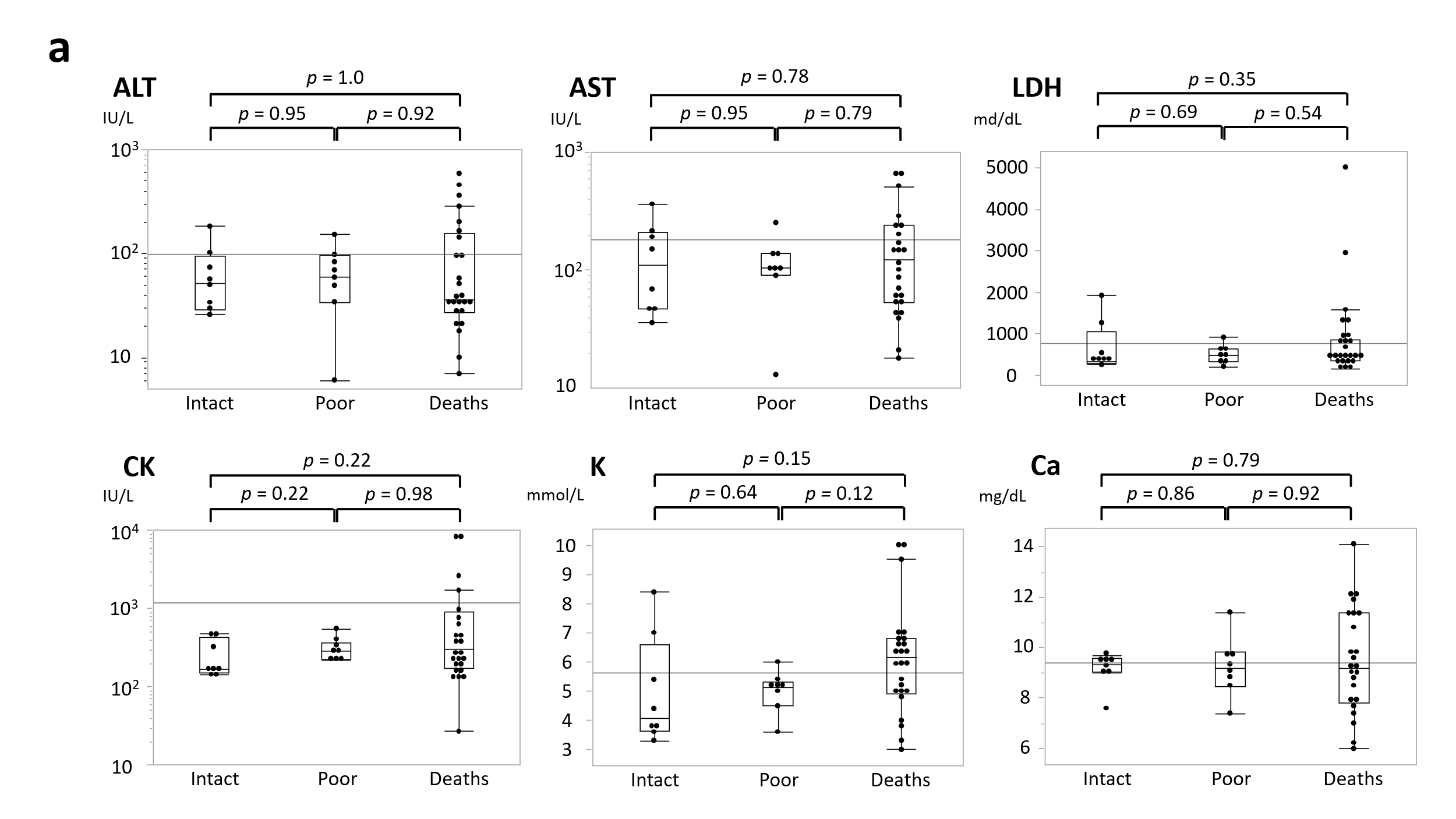


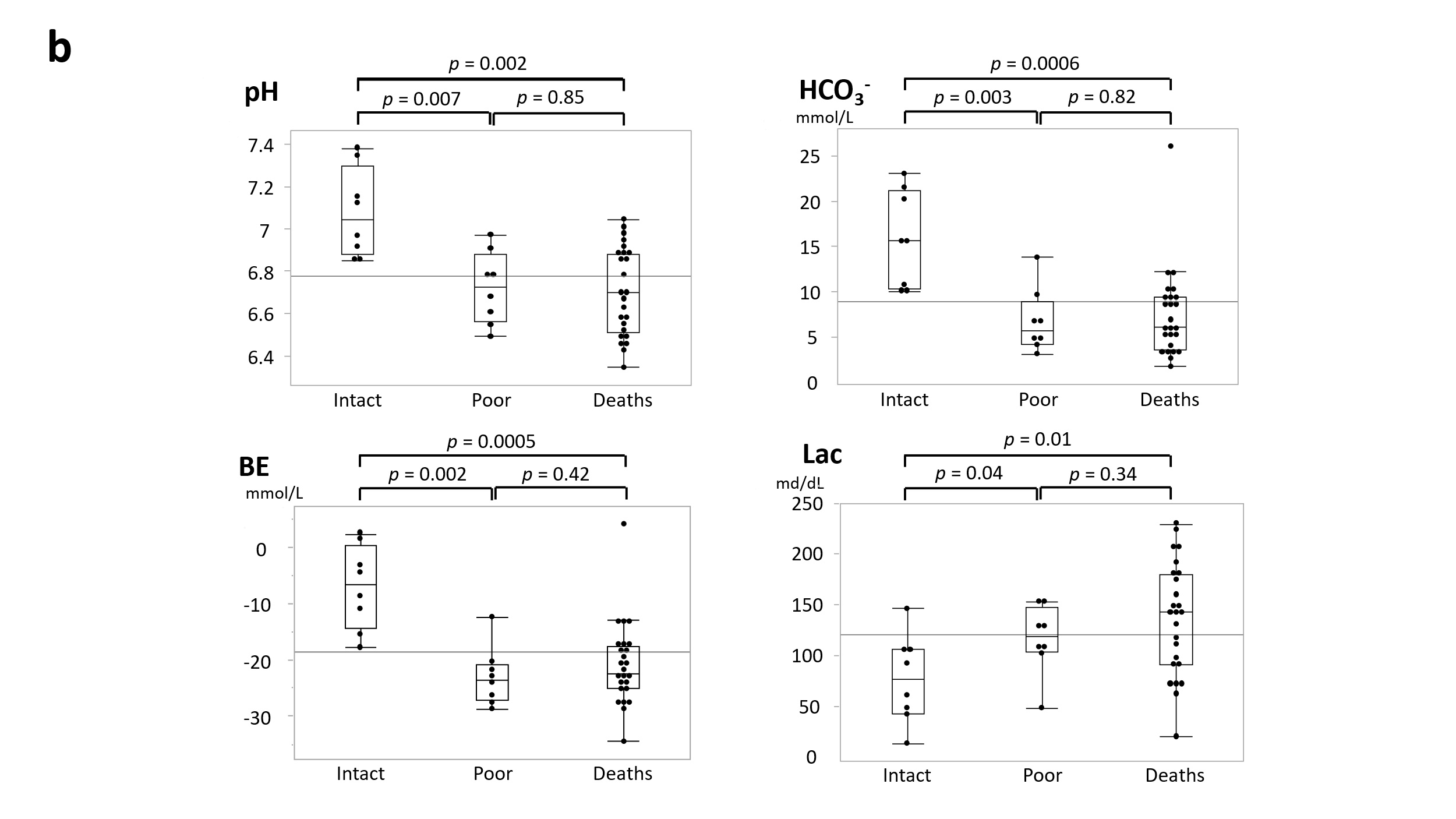


Initial biochemical values (a) and initial blood gas values (b) of 41 witnessed OHCA patients were compared between the two outcome groups. Data are shown as the median (line), upper and lower quartiles (box), and range (whiskers). The Mann-Whitney U-test *P*-values are shown above the bars. OHCA: out-of-hospital cardiac arrest.
